# The Need for Protocol-Based Training in Delivering Bad News in Dentistry: A Cross-Sectional Survey Among Dental Professionals in Jeddah, Saudi Arabia

**DOI:** 10.7759/cureus.52218

**Published:** 2024-01-13

**Authors:** Akram F Qutob

**Affiliations:** 1 Dental Public Health/Pediatric Dentistry, King Abdulaziz University, Jeddah, SAU

**Keywords:** delivering, dentistry, bad, news, breaking

## Abstract

Aim: This study aimed to explore the awareness and utilization of protocols (e.g., the SPIKES protocol) for delivering bad news among dental professionals and the perceived need for such training.

Methods: This study employed a cross-sectional design. A web-based self-administered questionnaire was distributed among dental students, general dentists, and dental specialists/consultants in Jeddah City through social media groups. The questionnaire included questions regarding the knowledge, attitudes, and practices of delivering bad news in dentistry and the use of the SPIKES protocol. Descriptive statistics and bivariate and multivariate analyses were performed to determine the research objectives.

Results: Two hundred and twelve participants responded to the questionnaire, with an almost equal distribution between genders. Specialists/consultants and dental students comprised 87.8% of the respondents. Most respondents (70%) were unaware of any protocols for delivering bad news, and approximately 89% were unaware of the SPIKES protocol. Only 7% reported using the SPIKES protocol to deliver bad news. The bivariate analysis revealed two significant associations. The first association indicated a variation in awareness based on professional status, and the second suggested that experience in delivering bad news affected perceptions of the importance of protocol-based training. Logistic regression models revealed that specialist/consultant status was significantly associated with decreased awareness of protocols (OR = 0.287, 95% CI 0.091-0.903) as compared to general dentists and that other variables, including sex and experience in delivering bad news, did not indicate a statistically significant effect.

Conclusion: Most dentists in Jeddah reported the need to be familiar with and practice delivering bad news in dentistry. They agreed that it would improve patients’ acceptance of bad news if it were performed in a systematic and organized manner (e.g., the SPIKES protocol).

## Introduction

Empathy is a fundamental skill for all healthcare professionals, as it facilitates good quality healthcare and improves communication between patients and caregivers [1]. Empathy requires a level of emotional intelligence based on self and social competence, by which an individual can recognize their feelings and know how to control them, recognize others’ feelings, and know how to interact with them. Elements of empathy include healthcare providers appreciating patients as humans with no judgment, seeing the world from patients’ eyes, understanding their feelings, and communicating that understanding [2].

In dentistry, as is the case in medicine, bad news can range from the need to perform a simple surgery to the diagnosis of an extreme condition such as cancer. The bad news is subjective from patient to patient; for example, a diagnosis of dental caries in a caring patient with no previous history of dental caries may be devastating compared to that in a patient with a high risk of dental caries. Delivering bad news is an essential skill that healthcare providers must acquire and practice for better patient care. Multiple protocols are used in healthcare to deliver bad news; however, the SPIKES protocol is often used because of its clarity and simplicity [3]. The SPIKES protocol is a well-known and validated tool used to deliver bad news. The protocol starts by making sure that the setting of the place where the bad news is being delivered is well chosen in a quiet private area, preventing distractors such as the doctor's phone or pager from being set on silent so as not to disturb the delivery of the news, and making sure that the doctor is setting at the patient's level not standing while delivering the news. This is followed by asking about the patient's perspective to increase the doctor's level of empathy. The patient's perspective includes asking questions about how the condition is affecting the patient physically and emotionally. Then the doctor should ask the patient about the preferred method for delivering the news e.g. if the patient prefers to listen directly from the doctor, or would like to have a patient relative or a friend present while delivering the news, or having the news delivered via a written report. The doctor should start giving some information about the condition and the treatment plan in brief and allow the patient to ask any questions related to the condition. The doctor throughout the interaction with the patient should pay special attention to clues about the patient's emotions and respond accordingly by physical gestures or verbal statements. At the end of the doctor-patient encounter, the doctor should ask the patient to repeat what he/she understood from the whole encounter and if the patient can't recall accurately what has been delivered, then the doctor should summarize again before the patient leaves. Few studies have assessed the knowledge, attitudes, and behaviors of dentists and dental students regarding delivering bad news [4]; hence, this study was conducted to find that gap in the literature.

This study aimed to explore the awareness and utilization of protocols (e.g., the SPIKES protocol) for delivering bad news among dental professionals and the perceived need for such training. The study objectives were to (1) explore the awareness of any protocol for delivering bad news in dentistry, including specific awareness of the SPIKES protocol; (2) find out the perceived importance of using a structured protocol-based training for delivering bad news among dental professionals; (3) investigate the actual use of SPIKES protocol or any other known protocols in delivering bad news in dentistry; (4) examine a difference in awareness, use, and perceived importance of protocols to deliver bad news including SPIKES protocol among different groups such as undergraduate/postgraduate dental students, general dentists, and dental specialists/consultants; and (5) check for any correlations between the awareness and use of protocols to deliver bad news in dentistry including SPIKES protocol with the perceived importance of such protocols.

## Materials and methods

This cross-sectional survey was conducted to investigate dental professionals’ awareness, applications, and perceived importance of protocols for delivering bad news. The participants included undergraduate and postgraduate dental students, general dentists, dental specialists, and consultants. A web-based self-administered questionnaire was developed, comprising sections on demographic information (sex and professional status), experience in delivering bad news, knowledge of delivering bad news protocols (including awareness of the SPIKES protocol), the practical use of these protocols, and perceptions regarding the necessity of training for delivering bad news in dentistry. SPIKES protocol was used due to its wide use in the medical field as well as for its clarity and ease of understanding and application compared to more complicated or simplified protocols. The online survey was developed via Google Forms and distributed to dental students, general dentists, and dental specialists/consultants in Jeddah, Saudi Arabia. The participants were reached through social media groups (WhatsApp) and word-of-mouth. The survey targeted dental students in both government and private universities as well as dentists working in government and private dental clinics and hospitals. The survey included a statement at the beginning of the survey explaining the purpose of the survey and stating that by choosing to participate in the survey the participant is consenting to be part of the study.

The questionnaire was pretested among a focus group of dental professionals including undergraduate/postgraduate students, general dentists, and dental specialists/consultants. The questionnaire was adjusted based on the responses from the focus group before its distribution. The questionnaire included the following variables: (1) whether participants were aware of any protocols for delivering bad news; (2) specific awareness of the SPIKES protocol; (3) whether participants had ever used the SPIKES protocol or any other protocol for delivering bad news; (4) attitudes toward the significance of using established protocols in delivering bad news; and (5) demographic and professional variables, including gender, professional status (undergraduate or postgraduate student, general dentist, and specialist/consultant), and experience in delivering bad news.

Sample size calculations were not possible, given the scarcity of similar topics to investigate. Therefore, convenience sampling was used to obtain responses from dental students and dentists in Jeddah. Data analysis was conducted in multiple phases using Python statistical software (version 3.10.4, Python Software Foundation, Wilmington, US). Descriptive statistics were used to display the frequency distributions and percentages that characterized the respondents' demographics, awareness, use, and perceptions of protocols for delivering bad news in dentistry. This was followed by bivariate analysis using chi-squared tests to examine associations between categorical variables, such as professional status, awareness, or the use of protocols for delivering bad news, and the perceptions of their importance. Multivariate logistic regression analysis was performed to determine the influence of multiple factors on the awareness and use of protocols for delivering bad news, as well as the perception of their importance. The logistic regression models provided coefficients, standard errors, z-scores, p-values, and odds ratios (OR) with 95% confidence intervals (CIs). Reference categories for categorical variables were specified for an accurate interpretation of the regression coefficients.

## Results

Descriptive statistics

Two hundred and twelve participants responded to the questionnaire. The responses were obtained from a diverse cohort of dental professionals, including undergraduate and postgraduate dental students, general dentists, and dental specialists and consultants. The majority of respondents were female (approximately 51%). Most respondents were dental specialists and consultants (47.2%), followed by dental students (40.6%), general dentists, and postgraduate students (7.1% and 5.1%, respectively).

Approximately 70% of the respondents were unaware of any protocols for delivering bad news, and approximately 89% were unaware of the SPIKES protocol. Only 7% reported using the SPIKES protocol, or any other known protocol, to deliver bad news. A significant majority (83%) believed that using an established protocol would improve patients’ acceptance of bad news.

Bivariate analysis

Chi-squared tests were conducted on the four sets of associations to test their significance. Two of the four associations were not significant (Tables 1-2).

**Table 1:** Bivariate Analysis of the Association Between the Awareness of the SPIKES Protocol and Study/Work Status.

Work Status	No	(%)	Yes	(%)	Total	(%)
Specialist/Consultant	89	89	11	11	100	100
Undergrad Dental Student	77	89.53	9	10.47	86	100
General Dentist	13	86.67	2	13.33	15	100
Postgrad Dental Student	10	83.33	2	16.67	12	100
Total	189	88.73	24	11.27	213	100
p-Value	0.924					

**Table 2:** Bivariate Analysis of the Association Between the Use of Protocols to Deliver Bad News and Study/Work Status.

Work Status	No	(%)	Yes	(%)	Total	(%)
General Dentist	13	86.67	2	13.33	15	100
Postgrad Dental Student	10	83.33	2	16.67	12	100
Specialist/ Consultant	95	95	5	5	100	100
Undergrad Dental Student	79	91.86	7	8.14	86	100
Total	197	92.49	16	7.51	213	100
p-Value	0.371					

The remaining sets revealed significant associations, namely the awareness of any protocols for delivering bad news versus study/work status, and the perceived importance of protocol-based training to deliver bad news versus the experience of delivering bad news. Results of the first comparison (the awareness of any protocol versus study/work status) revealed a significant association (χ² = 8.98, p = 0.0296), indicating variation in awareness based on professional status (Table 3).

**Table 3:** Bivariate Analysis of the Association Between the Awareness of any Protocol to Deliver Bad News and Study/Work Status.

Work Status	No		(%)	Yes	(%)	Total	(%)
General Dentist	8	53.33		7	46.67	15	100
Postgrad Dental Student	5	41.67		7	58.33	12	100
Specialist/Consultant	77	77		23	23	100	100
Undergrad Dental Student	59	68.6		27	31.4	86	100
Total	149	69.95		64	30.05	213	100
p-Value	0.0296						

In the second comparison (the perceived importance of protocol-based training versus the experience of delivering bad news), there was a statistically significant association (χ² = 9.77, p = 0.0075), suggesting that experience in delivering bad news impacts the perceptions of the importance of protocol-based training (Table 4).

**Table 4:** Bivariate Analysis of the Association Between the Perceived Importance of Protocol-Based Training to Deliver Bad News and the Experience of Delivering Bad News.

Experience	Unsure	(%)	No	(%)	Yes	(%)	Total	(%)
No	25	22.12	3	2.65	85	75.22	113	100
Yes	7	7	2	2	91	91	100	100
Total	32	15.02	5	2.35	176	82.63	213	100
p-Value	0.0075							

Multivariate analysis

Three logistic regression models were developed to further explore the relationships between predicting awareness of any protocol for delivering bad news in dentistry, the use of protocols including the SPIKES protocol, and the perceived importance of protocol-based training for delivering bad news in dentistry.

Model 1 (Predicting Awareness of Any Protocol)

Specialist/consultant status was significantly associated with decreased awareness of the protocols (OR = 0.287, 95% CI 0.091-0.903, p = 0.0328). Other variables, including gender and the experience of delivering bad news, did not exhibit statistically significant effects.

The analysis in Table 5 provides a clear understanding of how each predictor, relative to its reference category, influenced awareness of protocols in the field of dentistry.

**Table 5:** Logistic Regression Model to Examine the Prediction of Awareness of Any Protocol for Delivering Bad News in Dentistry.

Variable	Coefficient	Std. Error	z-Score	P-Value	Odds Ratio	95% CI Lower	95% CI Upper	Reference Category
const	-0.5961	0.5824	-1.0236	0.3060	0.5509	0.1759	1.7252	-
Gender	0.4156	0.3247	1.2800	0.2005	1.5153	0.8019	2.8632	Male
Experience Delivering Bad News	0.5103	0.3449	1.4796	0.1390	1.6659	0.8473	3.2752	No
Postgraduate Dental Student	0.5197	0.7904	0.6575	0.5109	1.6815	0.3572	7.9165	General Dentist
Specialist/Consultant	-1.2480	0.5846	-2.1349	0.0328	0.2871	0.0913	0.9028	General Dentist
Undergraduate Dental Student	-0.4641	0.5811	-0.7988	0.4244	0.6287	0.2013	1.9634	General Dentist

The OR for gender suggests that the odds of being aware of any protocol for delivering bad news in dentistry are 1.5 times higher for females than for males. The significant negative coefficient for specialists and consultants indicated that these professionals had lower odds of being aware of protocols than general dentists (reference category).

Model 2 (Predicting Use of Protocols Including SPIKES Protocol)

No significant predictors were identified for the use of protocols for delivering bad news, including the SPIKES protocol, suggesting that the factors considered in this study did not strongly influence the actual application of protocols to deliver bad news. Table 6 provides a clear understanding of how each predictor, relative to its reference category, influences the use of protocols, including the SPIKES protocol, in the field of dentistry.

**Table 6:** Logistic Regression Model to Examine the Prediction of Use of Protocols Including SPIKES Protocol to Deliver Bad News in Dentistry.

Variable	Coefficient	Std. Error	z-Score	P-Value	Odds Ratio	95% CI Lower	95% CI Upper	Reference Category
const	-2.1789	0.8936	-2.4383	0.0148	0.1132	0.0196	0.6522	-
Gender	-0.1385	0.5479	-0.2529	0.8004	0.8706	0.2975	2.548	Male
Experience Delivering Bad News	0.7074	0.5812	1.2172	0.2235	2.0287	0.6494	6.3375	No
Postgraduate Dental Student	0.2989	1.0953	0.2729	0.7849	1.3484	0.1576	11.5375	General Dentist
Specialist/Consultant	-1.2086	0.9038	-1.3373	0.1811	0.2986	0.0508	1.7556	General Dentist
Undergraduate Dental Student	-0.4027	0.8802	-0.4575	0.6473	0.6685	0.1191	3.7528	General Dentist

The model indicated that the overall predictors had a statistically significant effect on the likelihood of using protocols. Gender, experience delivering bad news, and study/work status did not exhibit statistically significant effects on the use of protocols. OR suggested the likelihood of using protocols compared to the reference categories, but none of the variables exhibited strong associations.

Model 3 (Predicting Perceived Importance of Protocol-Based Training)

Similar to the protocol use model (Model 2), no statistically significant predictors were identified, indicating that perceptions regarding the importance of protocol-based training were not strongly influenced by the variables included in this model. The analysis in Table 7 provides insights into the factors influencing awareness of and attitudes toward protocols for delivering bad news in dentistry.

**Table 7:** Logistic Regression Model to Examine the Prediction of Perceived Importance of Protocol-Based Training for Delivering Bad News in Dentistry.

Variable	Coefficient	Std. Error	z-Score	P-Value	Odds Ratio	95% CI Lower	95% CI Upper	Reference Category
const	1.0572	0.7117	1.4855	0.1374	2.8784	0.7134	11.6132	-
Gender	0.1209	0.4051	0.2985	0.7653	1.1285	0.5102	2.4963	Male
Experience Delivering Bad News	0.6273	0.4541	1.3814	0.1672	1.8726	0.7690	4.5602	No
Postgraduate Dental Student	1.0565	1.2337	0.8563	0.3918	2.8762	0.2562	32.2826	General Dentist
Specialist/Consultant	1.2256	0.7819	1.5674	0.1170	3.4062	0.7357	15.7707	General Dentist
Undergraduate Dental Student	-0.4489	0.7019	-0.6396	0.5224	0.6383	0.1613	2.5261	General Dentist

The CI and OR represent the effect of each predictor on the perceived importance of protocol-based training, with OR indicating the likelihood compared with the reference categories. None of the predictors had a statistically significant effect on the perceived importance of protocol-based training, as indicated by the p-values. OR provide an estimate of how much more (or less) likely respondents are to perceive protocol-based training as important relative to the reference category.

## Discussion

Awareness and application of delivering bad news protocols

A significant finding of this study is the low level of awareness and application of delivering bad news protocols, such as the SPIKES protocol, among dental professionals. This is consistent with previous studies indicating a general lack of awareness of communication protocols in various medical fields [5-7]. The underutilization of these protocols, as observed in our study, aligns with the findings of Adebayo et al. (2013), who reported similar trends in medical settings [5]. Despite its high perceived importance, its low usage rate underscores the educational gap that dental curricula can address [8].

Influence of professional status

This study revealed a significant association between professional status and awareness of protocols for delivering bad news. Specialists/consultants were less likely to be aware of these protocols than their counterparts were. This might be attributed to differences in educational backgrounds or a lack of continuous professional development focusing on communication skills [9]. Contrarily, undergraduate and postgraduate students exhibited relatively high awareness, possibly because of more recent exposure to educational content covering these aspects. However, contrary to our results regarding the awareness level and skills of undergraduate and postgraduate dental students, this skill is still lacking in other countries or possibly in other universities or training hospitals in Saudi Arabia yet to be discovered [10,11,8].

Perceived importance of protocol-based training

The high-perceived importance of protocol-based training in our sample reflects the growing recognition of the value of structured communication in patient care. This is supported by literature emphasizing the positive impact of protocol-based communication training on patient outcomes and satisfaction [12]. However, the lack of significant predictors of this perception suggests that other factors possibly related to personal attitudes or institutional culture may play a role.

Limitations and future directions

Our study had some limitations, including its cross-sectional design and reliance on self-reported data, which may have been subject to response bias. Additionally, the sample may not be representative of the broader dental professional community. Future research should focus on longitudinal designs to assess changes over time, particularly in response to targeted educational interventions.

## Conclusions

Despite a strong belief in the importance of protocol-based training, there is a significant gap in the awareness and usage of such protocols, especially the SPIKES protocol. These data suggest the need to integrate training on delivering bad news using established protocols in dental education, especially for undergraduate and postgraduate students. As these groups showed higher awareness, but lower usage, targeted training programs may be particularly beneficial. With the majority believing in the positive impact of such protocols on patient acceptance, there is a compelling argument for the widespread adoption of these practices in dental settings.
